# 2-Hydr­oxy-*N*′-[(*E*)-(3-hydr­oxy-2-naphth­yl)methyl­ene]benzohydrazide

**DOI:** 10.1107/S1600536808044073

**Published:** 2009-01-08

**Authors:** Yuying Sun, Hong-Gang Li, Xiao Wang, Shizhou Fu, Daqi Wang

**Affiliations:** a Analytical and Testing Center of Beihua University, Jilin 132013, People’s Republic of China; b Clinical Medicine Department, Weifang Medical University, Weifang, Shangdong 261042, People’s Republic of China; c Department of Chemistry, Liaocheng University, Liaocheng 250059, People’s Republic of China

## Abstract

In the title mol­ecule, C_18_H_14_N_2_O_3_, O—H⋯N and N—H⋯O hydrogen bonds influence the mol­ecular conformation; the benzene and naphthalene planes are inclined at a dihedral angle of 11.54 (5)°. In the crystal structure, inter­molecular O—H⋯O hydrogen bonds link the mol­ecules into chains running in the [01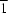] direction.

## Related literature

For useful applications of salicyloyl hydrazide derivatives, see: Sumita *et al.* (1999 ▶). For the crystal structure of (*E*)-2-hydr­oxy-*N*′-(3-hydr­oxy-4-methoxy­benzyl­idene)benzohydrazide, see: Luo (2007 ▶).
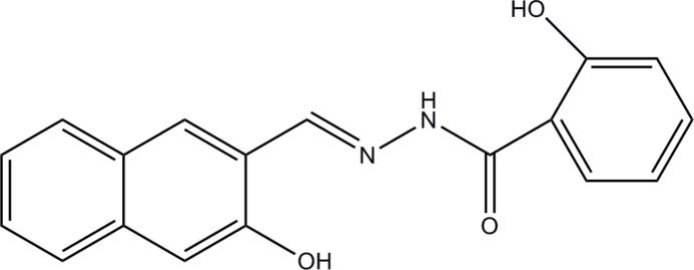


## Experimental

### 

#### Crystal data

C_18_H_14_N_2_O_3_
*M* *_r_* = 306.31
Orthorhombic, 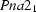

*a* = 21.124 (2) Å
*b* = 11.6212 (13) Å
*c* = 5.9826 (8) Å
*V* = 1468.6 (3) Å^3^
*Z* = 4
Mo *K*α radiation
μ = 0.10 mm^−1^
*T* = 298 (2) K
0.32 × 0.18 × 0.15 mm

#### Data collection

Bruker SMART CCD area-detector diffractometer
Absorption correction: multi-scan (*SADABS*; Sheldrick, 1996 ▶) *T*_min_ = 0.970, *T*_max_ = 0.986
6099 measured reflections
1422 independent reflections
896 reflections with *I* > 2σ(*I*)
*R*_int_ = 0.057

#### Refinement

*R*[*F* ^2^ > 2σ(*F* ^2^)] = 0.040
*wR*(*F* ^2^) = 0.076
*S* = 1.04
1422 reflections
209 parameters
1 restraint
H-atom parameters constrained
Δρ_max_ = 0.15 e Å^−3^
Δρ_min_ = −0.16 e Å^−3^

### 

Data collection: *SMART* (Siemens, 1996 ▶); cell refinement: *SAINT* (Siemens, 1996 ▶); data reduction: *SAINT*; program(s) used to solve structure: *SHELXS97* (Sheldrick, 2008 ▶); program(s) used to refine structure: *SHELXL97* (Sheldrick, 2008 ▶); molecular graphics: *SHELXTL* (Sheldrick, 2008 ▶); software used to prepare material for publication: *SHELXTL*.

## Supplementary Material

Crystal structure: contains datablocks I, global. DOI: 10.1107/S1600536808044073/cv2501sup1.cif

Structure factors: contains datablocks I. DOI: 10.1107/S1600536808044073/cv2501Isup2.hkl

Additional supplementary materials:  crystallographic information; 3D view; checkCIF report

## Figures and Tables

**Table 1:** Hydrogen-bond geometry (Å, °)

*D*—H⋯*A*	*D*—H	H⋯*A*	*D*⋯*A*	*D*—H⋯*A*
O3—H3⋯N2	0.82	1.90	2.623 (5)	146
N1—H1⋯O2	0.86	1.92	2.620 (4)	137
O2—H2⋯O1^i^	0.82	1.81	2.573 (4)	155

Symmetry code: (i) 
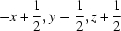
.

**Fig. 1.**
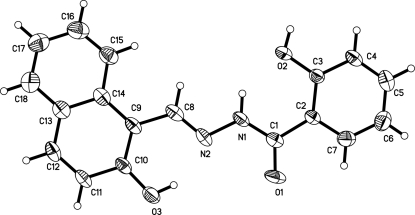
ORTEP drawing of the title molecule with atomic numbering scheme and displacement ellipsoids at 30% probability level.

## supplementary crystallographic information

### Comment

Salicyloyl hydrazide is an important organic intermediate, it can act as moulding board in inorganic complex (Sumita *et al.*, 1999). In this paper, we present the title compound (I), which was synthesized by the reaction of 2-hydroxyl naphthaldehyde and salicyloyl hydrazide.

In (I) (Fig. 1), the bond lengths and angles are normal and comparable to those observed in the reported compound (Luo, 2007). In the crystal structure, the C8=N2 bond length is 1.279 (5) Å showing the double-bond character. The dihedral angle between the naphthalene ring and C8/N2/N1 is 10.14 (3) Å, the C1/N1/N2 and benzene ring form a dihedral angle of 6.70 (4) Å showing that intramolecular O—H···N and N—H···O hydrogen bonds (Table 1) influence the molecular conformation.

In the crystal, intermolecular O—H···O hydrogen bonds (Table 1) link the molecules into chains running in direction [01–1].

### Experimental

Salicyloyl hydrazide (0.5 mmol) and freshly 2-hydroxyl naphthaldehyde (0.5 mmol) were mixed in 50 ml flash. After stirring 30 min at 353 K, the mixture then cooling slowly to room temperature and affording the title compound, then recrystallized from ethanol, affording the title compound as a green crystalline solid. Elemental analysis: calculated for C_18_H_14_N_2_O_3_: C 70.58, H 4.61, N 9.15%; found: C 70.53, H 4.55, N 9.24%.

### Refinement

All H atoms were placed in geometrically idealized positions (N—H 0.86, O—H 0.82 and C—H=0.93 Å) and treated as riding, with *U*_iso_(H) = 1.2 *U*_eq_ of the parent atom. In the absence of any significant anomalous scatterers in the molecule, the 1353 Friedel pairs were merged before the final refinement.

### Crystal data

**Table:**

C_18_H_14_N_2_O_3_	*D*_x_ = 1.385 Mg m^−^^3^
*M**_r_* = 306.31	Mo *K*α radiation, λ = 0.71073 Å
Orthorhombic, *P**n**a*2_1_	Cell parameters from 1119 reflections
*a* = 21.124 (2) Å	θ = 2.6–25.4°
*b* = 11.6212 (13) Å	µ = 0.10 mm^−^^1^
*c* = 5.9826 (8) Å	*T* = 298 K
*V* = 1468.6 (3) Å^3^	Block, yellow
*Z* = 4	0.32 × 0.18 × 0.15 mm
*F*(000) = 640	

### Data collection

**Table:**

Bruker SMART CCD area-detector diffractometer	1422 independent reflections
Radiation source: fine-focus sealed tube	896 reflections with *I* > 2σ(*I*)
graphite	*R*_int_ = 0.057
φ and ω scans	θ_max_ = 25.0°, θ_min_ = 1.9°
Absorption correction: multi-scan (*SADABS*; Sheldrick, 1996)	*h* = −24→24
*T*_min_ = 0.970, *T*_max_ = 0.986	*k* = −13→8
6099 measured reflections	*l* = −7→6

### Refinement

**Table:**

Refinement on *F*^2^	Primary atom site location: structure-invariant direct methods
Least-squares matrix: full	Secondary atom site location: difference Fourier map
*R*[*F*^2^ > 2σ(*F*^2^)] = 0.040	Hydrogen site location: inferred from neighbouring sites
*wR*(*F*^2^) = 0.076	H-atom parameters constrained
*S* = 1.04	*w* = 1/[σ^2^(*F*_o_^2^) + (0.0012*P*)^2^ + 0.7621*P*] where *P* = (*F*_o_^2^ + 2*F*_c_^2^)/3
1422 reflections	(Δ/σ)_max_ = 0.001
209 parameters	Δρ_max_ = 0.15 e Å^−^^3^
1 restraint	Δρ_min_ = −0.16 e Å^−^^3^

### Special details

**Table:**

Geometry. All e.s.d.'s (except the e.s.d. in the dihedral angle between two l.s. planes) are estimated using the full covariance matrix. The cell e.s.d.'s are taken into account individually in the estimation of e.s.d.'s in distances, angles and torsion angles; correlations between e.s.d.'s in cell parameters are only used when they are defined by crystal symmetry. An approximate (isotropic) treatment of cell e.s.d.'s is used for estimating e.s.d.'s involving l.s. planes.
Refinement. Refinement of *F*^2^ against ALL reflections. The weighted *R*-factor *wR* and goodness of fit *S* are based on *F*^2^, conventional *R*-factors *R* are based on *F*, with *F* set to zero for negative *F*^2^. The threshold expression of *F*^2^ > σ(*F*^2^) is used only for calculating *R*-factors(gt) *etc*. and is not relevant to the choice of reflections for refinement. *R*-factors based on *F*^2^ are statistically about twice as large as those based on *F*, and *R*- factors based on ALL data will be even larger.

### Fractional atomic coordinates and isotropic or equivalent isotropic displacement parameters (Å^2^)

**Table:**

	*x*	*y*	*z*	*U*_iso_*/*U*_eq_	
N1	0.22373 (16)	0.9395 (3)	0.7320 (6)	0.0514 (10)	
H1	0.2172	0.8713	0.7828	0.062*	
N2	0.19138 (18)	0.9753 (3)	0.5434 (6)	0.0536 (11)	
O1	0.27673 (14)	1.1075 (2)	0.7748 (6)	0.0703 (10)	
O2	0.25278 (12)	0.7725 (2)	1.0077 (6)	0.0574 (9)	
H2	0.2526	0.7107	1.0739	0.086*	
O3	0.16012 (13)	1.1067 (3)	0.2045 (6)	0.0664 (10)	
H3	0.1787	1.0892	0.3199	0.100*	
C1	0.2649 (2)	1.0079 (4)	0.8375 (8)	0.0504 (12)	
C2	0.2955 (2)	0.9611 (3)	1.0404 (8)	0.0447 (11)	
C3	0.2891 (2)	0.8497 (3)	1.1229 (7)	0.0441 (12)	
C4	0.3185 (2)	0.8179 (4)	1.3196 (8)	0.0544 (13)	
H4	0.3135	0.7435	1.3740	0.065*	
C5	0.3548 (2)	0.8948 (5)	1.4348 (9)	0.0672 (15)	
H5	0.3750	0.8718	1.5655	0.081*	
C6	0.3618 (2)	1.0054 (5)	1.3596 (10)	0.0718 (16)	
H6	0.3863	1.0578	1.4392	0.086*	
C7	0.3321 (2)	1.0378 (4)	1.1646 (10)	0.0614 (14)	
H7	0.3366	1.1130	1.1142	0.074*	
C8	0.1536 (2)	0.8994 (4)	0.4641 (8)	0.0546 (13)	
H8	0.1507	0.8288	0.5363	0.066*	
C9	0.1152 (2)	0.9189 (4)	0.2659 (8)	0.0493 (12)	
C10	0.1211 (2)	1.0193 (4)	0.1429 (8)	0.0495 (12)	
C11	0.0868 (2)	1.0370 (4)	−0.0549 (8)	0.0560 (13)	
H11	0.0914	1.1054	−0.1339	0.067*	
C12	0.0468 (2)	0.9542 (4)	−0.1312 (9)	0.0607 (14)	
H12	0.0255	0.9653	−0.2656	0.073*	
C13	0.0369 (2)	0.8510 (4)	−0.0089 (9)	0.0548 (13)	
C14	0.07102 (19)	0.8331 (4)	0.1920 (8)	0.0511 (12)	
C15	0.0568 (2)	0.7320 (4)	0.3138 (9)	0.0656 (15)	
H15	0.0780	0.7173	0.4470	0.079*	
C16	0.0128 (2)	0.6564 (5)	0.2396 (11)	0.0790 (19)	
H16	0.0044	0.5909	0.3239	0.095*	
C17	−0.0202 (2)	0.6734 (5)	0.0415 (12)	0.0771 (17)	
H17	−0.0500	0.6199	−0.0067	0.093*	
C18	−0.0082 (2)	0.7700 (4)	−0.0809 (9)	0.0686 (16)	
H18	−0.0301	0.7825	−0.2136	0.082*	

### Atomic displacement parameters (Å^2^)

**Table:**

	*U*^11^	*U*^22^	*U*^33^	*U*^12^	*U*^13^	*U*^23^
N1	0.053 (2)	0.058 (2)	0.043 (3)	0.005 (2)	−0.001 (2)	0.022 (2)
N2	0.051 (2)	0.070 (3)	0.040 (2)	0.016 (2)	0.000 (2)	0.017 (2)
O1	0.086 (2)	0.0454 (18)	0.079 (3)	0.0026 (17)	0.004 (2)	0.0291 (19)
O2	0.076 (2)	0.0427 (16)	0.053 (2)	−0.0038 (16)	−0.012 (2)	0.0166 (17)
O3	0.063 (2)	0.081 (2)	0.056 (3)	−0.0053 (18)	−0.0055 (19)	0.029 (2)
C1	0.052 (3)	0.049 (3)	0.050 (3)	0.007 (2)	0.006 (3)	0.012 (2)
C2	0.046 (3)	0.043 (3)	0.044 (3)	0.005 (2)	0.001 (2)	0.009 (2)
C3	0.050 (3)	0.042 (3)	0.040 (3)	0.000 (2)	−0.001 (2)	0.005 (2)
C4	0.060 (3)	0.058 (3)	0.045 (3)	0.008 (3)	0.001 (3)	0.017 (3)
C5	0.062 (3)	0.087 (4)	0.053 (4)	0.012 (3)	−0.010 (3)	0.003 (3)
C6	0.065 (3)	0.073 (4)	0.077 (4)	−0.006 (3)	−0.012 (3)	−0.011 (3)
C7	0.066 (3)	0.050 (3)	0.068 (4)	−0.002 (3)	0.002 (3)	0.007 (3)
C8	0.055 (3)	0.062 (3)	0.047 (3)	0.013 (3)	0.007 (3)	0.018 (3)
C9	0.047 (3)	0.063 (3)	0.037 (3)	0.011 (2)	0.005 (2)	0.014 (3)
C10	0.044 (3)	0.068 (3)	0.037 (3)	0.007 (2)	0.007 (3)	0.016 (3)
C11	0.054 (3)	0.075 (3)	0.039 (3)	0.011 (3)	−0.001 (3)	0.020 (3)
C12	0.059 (3)	0.088 (4)	0.035 (3)	0.009 (3)	0.000 (3)	0.007 (3)
C13	0.049 (3)	0.064 (3)	0.051 (3)	0.009 (3)	0.009 (3)	0.001 (3)
C14	0.049 (3)	0.059 (3)	0.046 (3)	0.017 (2)	0.006 (3)	0.004 (3)
C15	0.056 (3)	0.071 (3)	0.070 (4)	0.008 (3)	0.000 (3)	0.017 (3)
C16	0.069 (4)	0.068 (4)	0.100 (6)	0.003 (3)	0.001 (4)	0.018 (4)
C17	0.068 (4)	0.067 (4)	0.096 (5)	−0.003 (3)	0.002 (4)	0.001 (4)
C18	0.060 (3)	0.079 (4)	0.066 (4)	0.007 (3)	0.008 (3)	−0.008 (4)

### Geometric parameters (Å, °)

**Table:**

N1—C1	1.336 (5)	C8—C9	1.455 (6)
N1—N2	1.383 (5)	C8—H8	0.9300
N1—H1	0.8600	C9—C10	1.386 (5)
N2—C8	1.280 (5)	C9—C14	1.435 (5)
O1—C1	1.242 (5)	C10—C11	1.403 (6)
O2—C3	1.366 (5)	C11—C12	1.360 (6)
O2—H2	0.8200	C11—H11	0.9300
O3—C10	1.359 (5)	C12—C13	1.420 (6)
O3—H3	0.8200	C12—H12	0.9300
C1—C2	1.479 (6)	C13—C18	1.407 (6)
C2—C3	1.392 (5)	C13—C14	1.417 (6)
C2—C7	1.395 (6)	C14—C15	1.415 (6)
C3—C4	1.381 (6)	C15—C16	1.354 (6)
C4—C5	1.364 (6)	C15—H15	0.9300
C4—H4	0.9300	C16—C17	1.389 (7)
C5—C6	1.370 (7)	C16—H16	0.9300
C5—H5	0.9300	C17—C18	1.364 (6)
C6—C7	1.377 (7)	C17—H17	0.9300
C6—H6	0.9300	C18—H18	0.9300
C7—H7	0.9300		
			
C1—N1—N2	121.8 (4)	C10—C9—C14	118.7 (4)
C1—N1—H1	119.1	C10—C9—C8	120.9 (4)
N2—N1—H1	119.1	C14—C9—C8	120.4 (4)
C8—N2—N1	113.8 (4)	O3—C10—C9	122.7 (4)
C3—O2—H2	109.5	O3—C10—C11	115.7 (4)
C10—O3—H3	109.5	C9—C10—C11	121.6 (5)
O1—C1—N1	122.9 (4)	C12—C11—C10	120.1 (5)
O1—C1—C2	120.2 (5)	C12—C11—H11	120.0
N1—C1—C2	116.9 (4)	C10—C11—H11	120.0
C3—C2—C7	117.3 (4)	C11—C12—C13	121.1 (5)
C3—C2—C1	126.2 (4)	C11—C12—H12	119.5
C7—C2—C1	116.4 (4)	C13—C12—H12	119.5
O2—C3—C4	120.5 (4)	C18—C13—C14	120.4 (5)
O2—C3—C2	119.1 (4)	C18—C13—C12	120.4 (5)
C4—C3—C2	120.5 (4)	C14—C13—C12	119.1 (5)
C5—C4—C3	120.6 (5)	C15—C14—C13	116.8 (5)
C5—C4—H4	119.7	C15—C14—C9	123.8 (5)
C3—C4—H4	119.7	C13—C14—C9	119.4 (4)
C4—C5—C6	120.6 (5)	C16—C15—C14	121.1 (5)
C4—C5—H5	119.7	C16—C15—H15	119.5
C6—C5—H5	119.7	C14—C15—H15	119.5
C5—C6—C7	119.0 (5)	C15—C16—C17	122.1 (6)
C5—C6—H6	120.5	C15—C16—H16	118.9
C7—C6—H6	120.5	C17—C16—H16	118.9
C6—C7—C2	122.0 (5)	C18—C17—C16	118.8 (6)
C6—C7—H7	119.0	C18—C17—H17	120.6
C2—C7—H7	119.0	C16—C17—H17	120.6
N2—C8—C9	122.9 (4)	C17—C18—C13	120.8 (6)
N2—C8—H8	118.6	C17—C18—H18	119.6
C9—C8—H8	118.6	C13—C18—H18	119.6

### Hydrogen-bond geometry (Å, °)

**Table:**

*D*—H···*A*	*D*—H	H···*A*	*D*···*A*	*D*—H···*A*
O3—H3···N2	0.82	1.90	2.623 (5)	146
N1—H1···O2	0.86	1.92	2.620 (4)	137
O2—H2···O1^i^	0.82	1.81	2.573 (4)	155

Symmetry codes: (i) −*x*+1/2, *y*−1/2, *z*+1/2.
