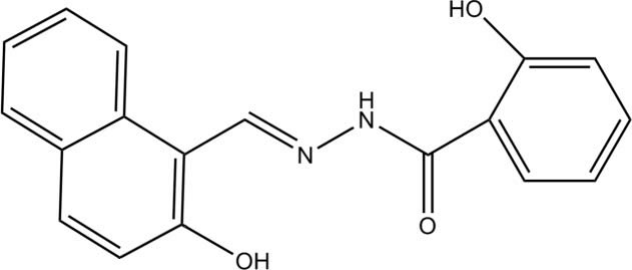# 2-Hydr­oxy-*N*′-[(*E*)-(3-hydr­oxy-2-naphth­yl)methyl­ene]benzohydrazide. Corrigendum

**DOI:** 10.1107/S1600536809023472

**Published:** 2009-07-31

**Authors:** Yuying Sun, Hong-Gang Li, Xiao Wang, Shizhou Fu, Daqi Wang

**Affiliations:** a Analytical and Testing Center of Beihua University, Jilin 132013, People’s Republic of China; b Clinical Medicine Department, Weifang Medical University, Weifang, Shangdong 261042, People’s Republic of China; c Department of Chemistry, Liaocheng University, Liaocheng 250059, People’s Republic of China

## Abstract

The chemical name of the title compound in the paper by Sun, Li, Wang, Fu & Wang [*Acta Cryst.* (2009), E**65**, o262] is corrected and the structural diagram is updated.

## Abstract 2

Corrigendum to *Acta Cryst.* (2009), E**65**, o262.

In the paper by Sun, Li, Wang, Fu & Wang [*Acta Cryst.* (2009), E**65**, o262], the chemical name given in the *Title* should be ‘2-Hydr­oxy-*N*′-[(*E*)-(2-hydr­oxy-1-naphth­yl)methyl­ene]­benzo­hydrazide’. An updated structural diagram is shown below.